# *ebv-sisRNA-3* contributes to the formation of G4-associated R-loop upstream of EBV lytic replication origin in latently infected cells

**DOI:** 10.1186/s13578-025-01437-3

**Published:** 2025-06-27

**Authors:** Bo Wang, Grace Tin Yun Chung, Yi Shuai, Man Wu, Danyang Ji, Raymond Wai Ming Lung, Yuk Yu Chan, Ming Ting Liu, Ee Ling Kong, Shin Yee Hui, Hei Man Leung, Qian Wu, Melissa Sue Ann Chan, Xin Wang, Guang Zhu, Kevin Y. Yip, Chun Kit Kwok, Kwok Wai Lo, Chi Man Tsang

**Affiliations:** 1 Department of Anatomical and Cellular Pathology, Prince of Wales Hospital, The Chinese University of Hong Kong, Hong Kong SAR, China; 2 Department of Chemistry and State Key Laboratory of Marine Pollution, City University of Hong Kong, Hong Kong SAR, China; 3 State Key Laboratory of Radiation Medicine and Protection, School of Radiation Medicine and Protection, Collaborative Innovation Center of Radiological Medicine of Jiangsu Higher Education Institutions, Soochow University, Suzhou, 215123 China; 4 Department of Surgery, The Chinese University of Hong Kong, Hong Kong SAR, China; 5 Institute for Advanced Study and State Key Laboratory of Molecular Neuroscience, Division of Life Science, The Hong Kong University of Science and Technology, Shatin, Hong Kong SAR China; 6 Department of Computer Science and Engineering, The Chinese University of Hong Kong, Shatin, New Territories, Hong Kong China; 7 Sanford Burnham Prebys Medical Discovery Institute, San Diego, La Jolla, CA 92037 USA; 8 Shenzhen Research Institute of City University of Hong Kong, Shenzhen, 518057 China; 9 State Key Laboratory of Translational Oncology, Sir YK Pao Centre for Cancer, The Chinese University of Hong Kong, Hong Kong SAR, China

**Keywords:** Epstein-barr virus, Nasopharyngeal carcinoma, ncRNA, sisRNA, R-loop, G-quadruplex

## Abstract

**Background:**

In EBV-associated epithelial cancers, only a limited number of viral proteins are translated, while multiple EBV-encoded non-coding RNAs are expressed to minimize activation of the host’s immune response. These non-coding RNAs have been shown to play regulatory roles in maintaining latency and promoting cancer progression while many aspects of them remain to be elucidated.

**Results:**

Here we revealed abundant expression of *ebv-sisRNA-3*, a novel EBV transcript in nasopharyngeal carcinoma and EBV-associated gastric cancer. This 5–7 kb non-polyA transcript is derived from *RPMS1* intron and is partially complementary to *LF3*. We observed high expression level of *ebv-sisRNA-3* in multiple EBV-positive cancer cells and clinical specimens, with accumulation in the cell nucleus. Notably, *ebv-sisRNA-3* invades the double-strand DNA *in trans* upstream of lytic replication origin in EBV genome and leads to the formation of R-loop and G-quadruplex simultaneously in the latently EBV-infected epithelial cells. Additionally, we revealed the locations of R-loops within the EBV genome and identified endogenous G-quadruplexes near the *EBER1* and *EBNA1* promoters.

**Conclusions:**

In this study, we revealed and characterized a novel EBV transcript *ebv-sisRNA-3* widely expressed in latently infected cells. The unique *ebv-sisRNA-3*-binding R-loop and G-quadruplex structures near lytic replication origin may play a significant role in EBV lytic replication.

**Supplementary Information:**

The online version contains supplementary material available at 10.1186/s13578-025-01437-3.

## Background

Epstein-Barr virus (EBV), also known as human herpesvirus 4 (HHV-4), belongs to the gammaherpesvirus family. EBV infection associated with various human lymphoid malignancies (Burkitt’s lymphoma, large B-cell lymphoma, Hodgkin disease and NK/T cell lymphomas) and epithelial cancers (nasopharyngeal carcinoma and gastric cancer) [1]. For EBV-associated epithelial cancers, EBV contributes to the development of all nonkeratinizing nasopharyngeal carcinoma (NPC) and 10% of gastric cancer (GC) of reported cases [1, 2]. EBV has two distinct life cycles, latency and lytic replication, in the infected cells. During the latent phase, only a small fraction of viral genes and non-coding RNAs (ncRNAs) are expressed, with the types of latently expressed genes varying depending on the cell type infected [3]. In EBV-associated gastric cancer (EBVaGC) and NPC, the type I and type II latency are typically established respectively, expressing the EBNA1 and latent membrane proteins, as well as multiple viral ncRNAs including the *EBERs*, and transcripts from the *BamHI A rightward transcript* (*BART*) locus [4, 5]. Plenty of ncRNAs are produced from *BART* locus, including the long non-coding RNA (lncRNA) *RPMS1* and two clusters of viral microRNAs (miRNAs) derived from the introns [6, 7, 8, 9].

Stable intronic sequence RNA (sisRNA) is relatively a new class of ncRNAs. They are defined as intronic sequences produced during splicing, and are not degraded immediately in cells [10]. The sisRNAs have been found across diverse species, and related in gene expression regulation and RNA splicing [11, 12, 13]. Two small EBV sisRNAs, *ebv-sisRNA-1* and *ebv-sisRNA-2*, generated from the W repeats in EBV genome, were identified in the B cells with EBV latency III infection [14]. The *ebv-sisRNA-1* is the third most abundant EBV encoded RNAs with ~ 21% abundance of *EBER1* [15]. Nevertheless, the function of *ebv-sisRNA-1* and *ebv-sisRNA-2* remains unclear.

There are two copies of lytic DNA replication origin, termed as *OriLyt* (*OriLyt* L and *OriLyt* R) in EBV genome [16]. *OriLyt* contains BZLF1-responsive elements (ZREs) and binding sites for the Sp1 and ZBP-89 proteins, which interact with the core viral replication proteins including the DNA polymerase processivity factor BMRF1 and the DNA polymerase BALF5 [17, 18]. Besides, the *OriLyt* L is colocalized with the promoter of the viral *BHLF1* gene [19]. Two G-rich sequences, the internal repeat 2 (*IR2*) and internal repeat 4 (*IR4*), are closely adjacent to the upstream of *OriLyt* L and *OriLyt* R, respectively.

R-loops are naturally formed during transcription, DNA replication and DNA repair, playing important roles in gene expression regulation and chromatin structure [20]. Early studies revealed that R-loop was intermediate for replication initiation at the origin in bacteriophage T4, *Escherichia coli* (*E. coli*) and human mitochondrial DNA [21, 22, 23, 24, 25]. In these models, R-loops are thought to form at replication origins, where they are subsequently processed by RNase H1 to generate 3′-ends that serve as primers for DNA synthesis. In EBV, it was reported that the R-loop at EBV *OriLyt*, was formed 3–4 h after lytic induction and essential for lytic replication initiation [26]. How the R-loop formed in *OriLyt* contributes to the initiation of lytic replication remains for further investigation.

By ChIP-seq assays, it was demonstrated that the G-quadruplex (G4) and R-loop were highly overlapped genome-wide in mouse embryonic stem cells [27]. The G4 motif, which could potentially form a G4 structure, is present in more than 60% of metazoan origins including mouse and human [28, 29]. Additionally, G4 motifs were reported to locate at upstream of the replication start site and were supposed to determine the precise position of replication initiation at a subset of origins [28, 30, 31]. Experimental evidence in chicken and mouse cells indicated the G4 motif is positively related with the replication origin activity [30, 31]. Notably, G4 helicase RecQ1 was found to interact with *OriLyt* of Kaposi’s sarcoma-associated herpesvirus (KSHV) during lytic replication [32].

In this study, we reported and characterized a novel EBV long non-polyA transcript and designated it as *ebv-sisRNA-3*. We showed that it is 5–7 kb in length and derived from *BART* region, part of which is complementary to the lytic transcript *LF3*. Notably, *ebv-sisRNA-3* is ubiquitously expressed in EBV-positive NPC and EBVaGC samples. We further revealed that the *ebv-sisRNA-3* invaded the double strand DNA and formed a DNA-RNA hybrid in *IR4* region, upstream of *OriLyt* R. Simultaneously, we detected G-quadruplex formation at the substituted complementary strand using both in vivo and in vitro methods. The formation of G4s and R-loop structures near *OriLyt* R associated with *ebv-sisRNA-3* during EBV latent stage is a potential novel mechanism for regulation of EBV lytic replication.

## Methods

### Cell lines

C666-1, HK1, HK1-EBV, SNU719, YCCEL1, AGS, LCL-1, LCL-2 and Raji cells were grown in RPMI 1640 medium (Cat. No. 31800022, ThermoFisher) with 10% Fetal bovine serum (ThermoFisher) [33, 34, 35, 36]. Raji cells were additionally supplemented with 2 mM L-glutamine (ThermoFisher). NPC43 was grown in RPMI 1640 medium (ThermoFisher) with 10% Fetal bovine serum (ThermoFisher), 1% glutamate and 10 µM ROCK inhibitor (Y27632) (Sigma-Aldrich). The immortalized nasopharyngeal epithelial cell lines NP550, NP460, and NP361 were grown in the medium prepared by mixing the Defined Keratinocyte SFM (with the Growth Supplement) (ThermoFisher) and EpiLife™ Medium (with 60 µM calcium) (ThermoFisher) in 1:1 ratio [37, 38, 39]. SNU719 cell line with inducible CRISPR dCAS9 system for BZLF1 activation was established by our lab as described previously and grown under same condition as unmodified SNU719 [40]. The U2OS cell line was grown in DMEM (ThermoFisher) with 10% Fetal bovine serum (ThermoFisher).

### Xenografts and tumor specimens

NPC xenografts X32, X47, X76, X2117, C15, X111, X113 and X666 were grafted in athymic mice [33, 37, 41, 42]. NPC and GC tumor specimens were from patients admitted to Prince of Wales Hospital, The Chinese University of Hong Kong.

### Ribosomal RNA-depleted RNA sequencing and poly(A)-captured RNA sequencing

Total RNA of SNU719, xenografts C15 and X666-1 was extracted using TRIzol reagent (ThermoFisher) following the manufacturer’s instructions. RNA samples from each cell line or xenograft were applied to poly(A)-captured RNA sequencing and Ribosomal RNA-depleted RNA sequencing, respectively. For poly(A)-captured RNA sequencing, mRNA was purified from total RNA using poly-T oligo-attached magnetic beads during library preparation. For Ribosomal RNA-depleted RNA sequencing, ribosomal RNA was removed from total RNA during library preparation. The library preparation and sequencing were performed on Illumina platform by Novogene company (Beijing, China).

### Northern blot

The DNA templates for generating *ebv-sisRNA-3* and *GAPDH* probes in in vitro transcription were amplified using KAPA2G Fast HotStart PCR Kit (Roche). The reaction was denatured at 95˚C for 5 min and amplified for 35 cycles with a 15 s denaturation at 95˚C and a 15 s annealing at 60˚C and a 15 s extension at 72˚C, followed by final extension at 72 °C for 1 min. For *ebv-sisRNA-3*, the template for PCR reaction was synthesized by company (IGE, Guangzhou, China). For GAPDH, PCR reaction was conducted with C666-1 cDNA as template. Sequence information for primers and synthesized DNA templates are listed in Table S1. PCR products were then purified and applied to in vitro transcription. The biotin-labeled *ebv-sisRNA-3* and *GAPDH* probes were generated by in vitro transcription using HiScribe^®^ T7 High Yield RNA Synthesis Kit (New England Biolabs) and Biotin-14-CTP (ThermoFisher) according to the manufacturer’s protocol. Total RNA samples of Xenograft C15, and cell lines C666-1, SNU719, NP460 were extracted with TRIzol reagent (ThermoFisher). For each sample, 10 µg total RNA was applied to Northern blot using the NorthernMax™ Kit (ThermoFisher) following the manufacturer’s instruction. The biotinylated RNA probes were diluted to 0.2 nM and hybridized with the RNAs crosslinked on the membrane at 73 °C for *ebv-sisRNA-3* probe (68 °C for *GAPDH* probe) overnight. After hybridization, the probes were washed twice with low stringency wash solution at room temperature followed by washing with high stringency buffer twice at 73 °C for *ebv-sisRNA-3* probe (68 °C for *GAPDH* probe) for 15 min. The blot with bound biotinylated RNA probes were detected using the Chemiluminescent Nucleic Acid Detection Module Kit (ThermoFisher).

### Rapid amplification of cDNA ends (RACE)

The total RNA of xenograft C15 was extracted with TRIzol reagent (ThermoFisher). 5’ and 3’ RACE assays were conducted using the SMARTer^®^ RACE 5’/3’ Kit (TaKaRa) following manufacturer’s instruction. All PCR products from 5’ and 3’ RACE assays were applied to TA cloning (pGEM^®^-T Easy Vector Systems) (Promega) followed by Sanger sequencing to identify the 5’ and 3’ ends. Primers for RACE-PCR reactions are listed in Table S2.

### RNAscope RNA in-situ hybridization (RISH)

Formalin-fixed paraffin-embedded cell pellets or tumor specimens were cut into 4 μm sections. Sample preparation and pretreatment were carried out following the user manual of ACDBio company, that is available at https://acdbio.com/documents/product-documents. RNAscope RISH assays were conducted using RNAscope 2.5 HD Reagent Kit—BROWN (ACDBio) according to the manufacturer’s protocol with minor modification. DNase I (New England Biolabs) (1:5 diluted with 1× DNase I Reaction Buffer) were added, incubated at 37 °C for 30 min and rinsed twice with distilled water before Protease Plus incubation for EBV targets. Probes targeting the *ebv-sisRNA-3* (Cat. No. 434071, ACDBio), *LF3* (Cat. No. 434061, ACDBio), *RPMS1* (Cat. No. 488631, ACDBio), *EBER1* (Cat. No. 310217, ACDBio), *BMRF1* (Cat. No. 450451, ACDBio) and *BZLF1* (Cat. No. 450411, ACDBio) were purchased from ACDBio company.

### RNAscope RISH combined with immunofluorescence staining

RNAscope RISH steps were conducted using Multiplex fluorescent reagent kit v2 (ACDBio) according to the manufacturer’s protocol with minor modification. For targeting *ebv-sisRNA-3*, DNase I (New England Biolabs) (1:5 diluted with 1× DNase I Reaction Buffer) were added, incubated at 37 °C for 30 min and rinsed twice with distilled water before Protease Plus incubation. For RNase A treatment, 1 mg/ml RNase A (1:100 dilution) were added and incubate during the DNase I digestion step. Probes targeting the *ebv-sisRNA-3* (Cat. No. 434071, ACDBio) were purchased from ACDBio company. After stopping by the HRP blocker step, the slides were subjected to Immunofluorescence (IF) staining for Lamin B1 detection using Lamin B1 antibody (1:1000). The *ebv-sisRNA-3* and Lamin B1 signals were developed using Opal 520 (1:1000 dilution) and Opal 570 fluorophore (1:500 dilution) (AKOYA Biosciences), respectively. Finaly, the stained cells were counterstained with DAPI and mounted. Imaging was performed using a LSM880 Laser Confocal Microscope with Zen software (Carl Zeiss, Germany). Staining score of RNAScope for *ebv-sisRNA-3* and *RPMS1* are listed in Table S3.

### DRIP-seq, DRIPc-seq and DRIP-qPCR

The DNA: RNA ImmunoPrecipitation (DRIP) for the C666-1, SNU719 and YCCEL1 cells were conducted following the protocol as described by Sanz et al. [43]. For DRIP-seq, the library preparation and sequencing were performed on Illumina platform by Novogene company (Beijing, China). For DRIPc-seq, the library preparation was performed with Swift RNA Library Kit (Swift Biosciences) and sequencing was performed on Illumina platform by Novogene company (Beijing, China). For DRIPc-Seq data analysis, raw sequencing reads were initially assessed for quality using FastQC (Version 0.12.1) and trimmed to remove adapters and low-quality bases with Cutadapt (Version 4.4). High-quality reads were aligned to the EBV genome using HISAT2 (Version 2.2.1) with the *--rna-strandness* parameter to specify RNA strandness. Strand-specific reads (sense and antisense) were normalized and assigned to their respective strands using bamCoverage (Version 3.5.1), generating strand-specific bigWig files for downstream visualization. For DRIP-seq analysis, sequencing reads were aligned to the EBV genome using Bowtie2 (Version 2.2.5). Duplicate reads were identified and marked with Picard (Version 3.0) to reduce potential biases in downstream analyses. Enriched R-loop regions were identified through peak calling using MACS2 (Version 2.2.9.1). DRIP-qPCR was performed with eluted DNA from DRIP assays using the *Power* SYBR™ Green PCR Master Mix (Applied Biosystems). Primers used in DRIP-qPCR are listed in Table S4.

### Potential quadruplex-forming sequence (PQS) prediction

The PQS prediction was conducted with G4Hunter using the following parameters: window size = 25, threshold = 1.5. The sequences of the whole EBV genome (sequence ID: KC207813.1, NCBI Nucleotide database) and *IR4* were uploaded for PQS prediction. Predicted PQS were presented with Integrative Genomics Viewer.

### Circular dichroism (CD) study

The oligos for Circular dichroism (CD) study were synthesized by Guangzhou IGE Biotechnology. The sequences of oligos are listed in Table S5. Before scanning, 2 ml of 5 µM oligos in buffer containing 10 mM Tris-HCl (pH 7.4), 100 mM KCl were annealed through heating at 95 °C for 5 min followed by slowly cooling to room temperature. CD spectra was detected on a Jasco CD J-1500 spectrometer (JASCO, Japan) using 1 cm path length quartz cuvette. The CD ellipticity (measured in millidegrees (mdeg)) from 220 to 320 nm at 1 nm interval was accumulated.

### BG4 purification

The plasmid pSANG10-3 F-BG4 was established by Shankar Balasubramanian and obtained from Addgene Addgene). The pSANG10-3 F-BG4 was transformed to *E. coli* BL21(DE3) strain for anti-G4 antibody (BG4) expression and purification. Cells in 500 ml medium were grown at 37 °C with shaking at 250 rpm to mid-log phase (OD600 nm approximately 0.5). The cells were then added with 0.1 mM IPTG (Cat. No. I6758, Sigma-Aldrich) to induce the BG4 expression and further grown at 25 °C for another 12 h. For BG4 purification, cells were sonicated, and the lysate was centrifuged at 12,000 g for 10 min to obtain the supernatant. The supernatant containing BG4 was further clarified using a 0.45 μm syringe filter and applied to a Ni-NTA 6FF (His-Tag) PrePacked Gravity Column (Sangon Biotech). The binding buffer containing 20 mM imidazole (Sangon Biotech) was used for washing and the elution buffer containing 250 mM imidazole was used for protein elution. The elution buffer was then replaced with PBS buffer using a centrifugal concentrator (Millipore) (10 kDa MWCO, 0.5 mL sample volume).

### Enzyme-linked immunosorbent assay (ELISA)

ELISA assay was performed to assess BG4 affinity using the Streptavidin Coated ELISA Plate (ThermoFisher) following the manufacturer’s instructions. Oligo sequences in ELISA assay are listed in Table S6. Briefly, the 50 nM biotinylated DNA oligos were annealed through heating at 95 °C for 5 min followed by slowly cooling to room temperature in 10 mM Tris HCl, pH 7.4, 100 mM KCl buffer and incubated in the streptavidin coated plate. The BG4 antibody with 8 gradient concentrations (40, 10, 2.5, 0.625, 0.15625, 0.0390625, 0.009765625, and 0 nM), were then incubated with each oligo. All reactions were tested in duplicate. the ANTI-FLAG^®^ M2 antibody (Merck) (1:2000), Anti-mouse IgG, HRP-linked Antibody (Cell Signaling Technology) (1:2000) and 100 µl TMB substrate solution (Cat. No. 002023, ThermoFisher) were applied for detecting the binding affinity after BG4 incubation. The reactions were then stopped by adding an equal amount of 1 M hydrochloric acid (HCl) to the reaction solution. The absorbance at OD_450 nm_ was read with the SpectraMax iD3 Microplate Reader (Molecular Devices, USA). Dissociation constants and binding curves were calculated with GraphPad Prism (GraphPad Software, Inc.) using the “Specific binding with Hill slope” equation.

### G4 ChIP-qPCR

The C666-1, SNU719 and YCCEL1 cells were collected at 70% confluency and fixed with 1% formaldehyde for 9 min. The G4 ChIP assays and data analysis were conducted following the published protocol as described by Robert Hänsel-Hertsch, et al. [44]. ChIP-qPCR was performed in triplicate with eluted DNA from ChIP assays using the *Power* SYBR™ Green PCR Master Mix (ThermoFisher). Primers used in G4 ChIP-qPCR are listed in Table S7.

### EBV DNA copy number analysis by qPCR

SNU719 cells with inducible CRISPR dCAS9 system for BZLF1 activation were incubated with 25 or 50 µM of TMPyP2 or TMPyP4 for 48 h, lytic induction was then triggered by treatment with 1 µg/ml doxycycline. Total gDNA was then extracted using the QIAamp DNA Mini Kit (250) (QIAGEN). To determine the relative EBV copy number, Ct value of EBV BamHI-W fragment and human genomic DNA in *LEPTIN* region were measured. The Ct value of EBV BamHI-W fragment was determined by qPCR using the TaqMan Universal PCR Master Mix (ThermoFisher). The Ct value of human genomic DNA in *LEPTIN* gene was determined by qPCR using the Power SYBR™ Green PCR Master Mix (ThermoFisher). The relative EBV copy number was determined by the Ct value of EBV BamHI-W fragment normalized to the Ct value of human genomic DNA in *LEPTIN* region by the delta-delta Ct method. Primers and probes used in qPCR are listed in Table S8.

## Results

### A novel non-poly(A) EBV transcript is derived from a BART intron

**Fig. 1:**
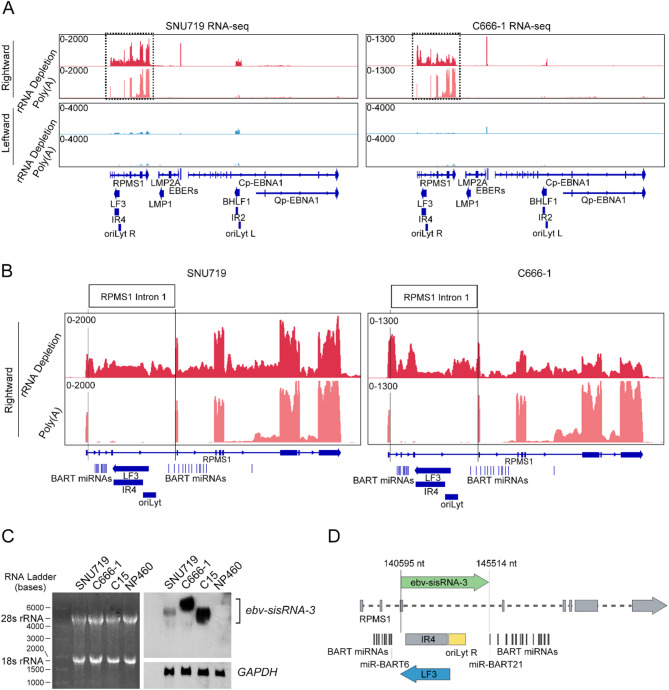
A novel non-poly(A) EBV transcript is derived from the *BART* intron. (**A**) Integrative Genomics Viewer (IGV) view for EBV transcriptome from both rRNA-depleted RNA-seq and poly(A) RNA-seq of SNU719 and C666-1 cells. The rightward and leftward EBV transcripts are shown in red and blue, respectively. Peaks of *BART* transcripts are highlighted by dotted line boxes. (**B**) The boxed region in (A) providing a closer view of the peaks for *BART* transcripts. (**C**) The *ebv-sisRNA-3* was detected by Northern blot as bands with about 5–7 kb bases in length (upper right panel). The 28S rRNA, 18S rRNA (left panel) and the 1.27 kb *GAPDH* (bottom right) transcript were served as loading controls. (**D**) The accurate 5’ and 3’ ends of *ebv-sisRNA-3* transcript were determined by RACE assays. The *ebv-sisRNA-3* mainly started between the *miR-BART6* and *IR4* region and stopped at the 5’ upstream of *miR-BART21* (EBV genomic position 14059–145514; Reference genome: NC_007605.1, NCBI Nucleotide database)

To comprehensively investigate the EBV transcriptome in epithelial cells, we have conducted RNA sequencing on both poly(A)-captured and ribosomal RNA-depleted RNA libraries of an EBVaGC cell line SNU719 and NPC cell line C666-1. The results indicated that multiple *BART* transcripts were consistently detected in the two cell lines by all sequencing study (Fig. 1A). Notably, in these EBV-positive epithelial cancer cell lines, we observed a significant number of reads within the intron 1 region of *RPMS1* in the EBV transcriptome derived from rRNA-depleted RNA-sequencing (rRNA-depleted RNA-seq), but not in the poly(A)-captured RNA-sequencing (poly(A) RNA-seq) (Fig. 1B). The finding suggests that a rightward non-poly(A) transcript derives from the EBV genome region at around nt138500 to nt146200 that is overlapped with the *IR4* region. This non-poly(A) transcript also overlaps with the antisense strand of the lytic *LF3* transcript. In early study, two non-poly(A) transcripts, *ebv-sisRNA-1* and *ebv-sisRNA-2* were reported in the EBV- latency III -transformed B cells. Nevertheless, their expression is rarely found in the EBV-positive NPC and GC cells (Figure S1).

To confirm the expression of this non-poly(A) transcript in EBV-positive epithelial cancers, we have designed a specific RNA probe targeting the rightward transcript in *IR4* region for Northern blot analysis in EBV-positive and EBV-negative samples. As shown in Fig. 1C and Figure S2, specific signals were detected in the SNU719 and C666-1 cell lines and an NPC xenograft C15, but not in the EBV-negative immortalized nasopharyngeal epithelial cell lines NP460. It is noted that the size of the transcript in C666-1 > 6 kB, while smaller size bands of 5–6 kB were observed in SNU719 and C15. The variation in length of the transcripts is attributable to the different repeat numbers in the *IR4* region of EBV genomes in these NPC samples [45]. The *IR4* repeat number in the EBV genome of EBV-infected B- and epithelial cells ranged from 22 to 34 [45]. It was reported that C15 contains only approximately 23 *IR4*-repeats, while the EBV genome in other NPC xenografts harbors 23 to 28 repeats in the *IR4* region [45]. We also conducted the rapid amplification of cDNA ends (RACE) assays to determine the 5’ and 3’ end of this non-poly(A) transcript in the NPC xenograft C15. By sequencing the PCR products of RACE assays, we revealed that the transcript started between the *miR-BART*6 and *IR4* region and stopped at the 5’ upstream of *miR-BART21*, overlapping with the EBV genome regions of *IR4*, *LF3* and *OriLyt* R (Fig. 1D, S3). In consistent to our current finding, Edwards et al. have previously reported the residual intronic pieces derived from processing of the pre-miRNAs in the intron of *RPMS1* in NPC tumors [46]. Taken together, we suggested that this non-poly(A) transcript is a EBV latent gene product commonly accumulated in EBV-positive epithelial cancers. This transcript originates from the *RPMS1* intron yet stably accumulated in the cells—unlike typical intronic sequences that are spliced out and rapidly degraded—it fits the definition of a sisRNA. We therefore defined it as a new EBV-encoded sisRNA and designated it as *ebv-sisRNA-3*.

### Detection of ebv-sisRNA-3 in NPC and EBVaGC by RNA in-situ hybridization (RISH)

**Fig. 2:**
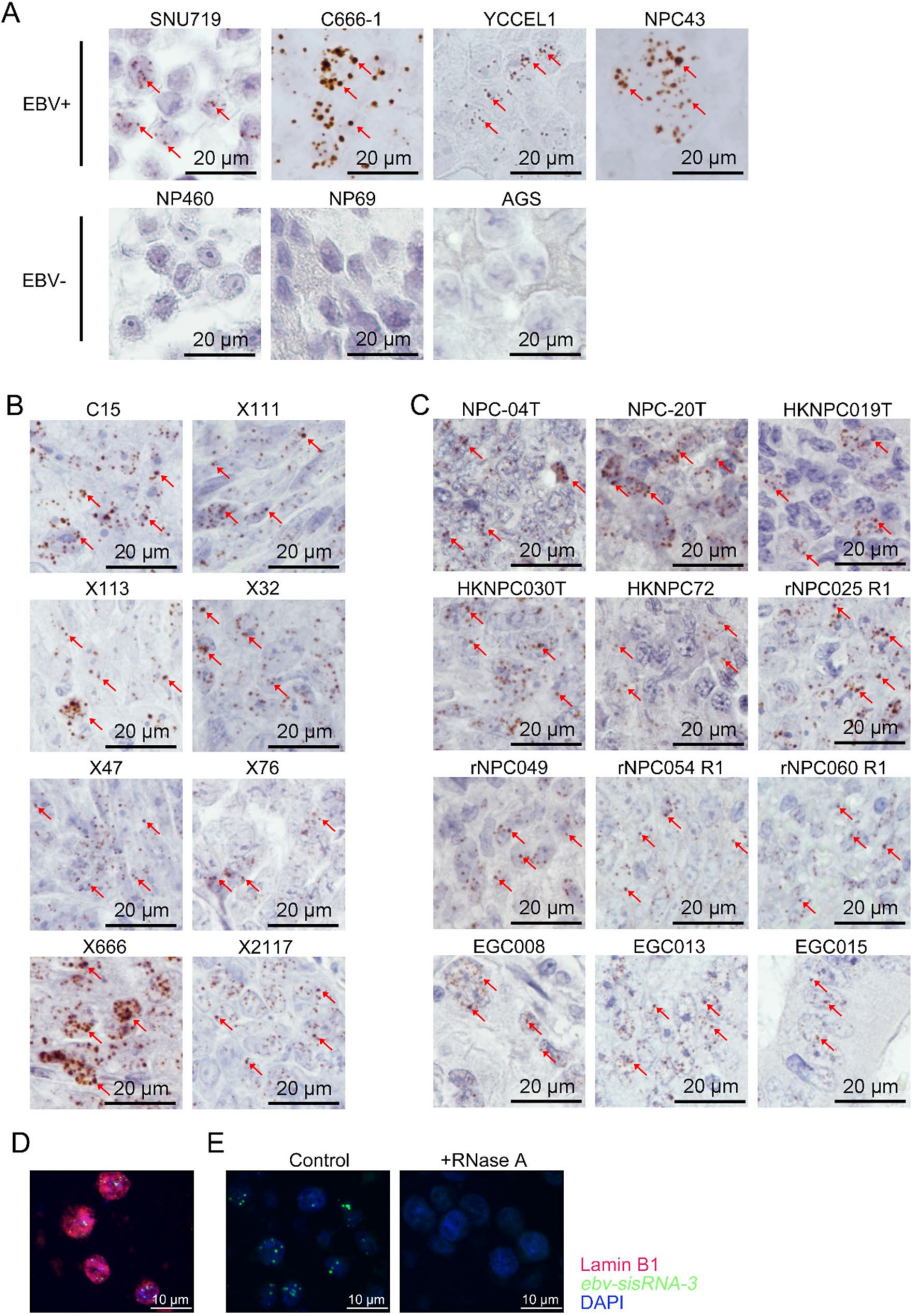
Detection of *ebv-sisRNA-3* in NPC and EBVaGC by RNA in-situ hybridization (RISH) analysis. RNAscope RISH analysis of *ebv-sisRNA-3* in EBV-positive and EBV-negative cell lines (**A**), NPC xenografts (**B**) and NPC and EBVaGC tumor specimens (**C**). Scale bars represent 20 μm. Representative signals of *ebv-sisRNA-3* are indicated with red arrows. (**D**) Co-staining of the nuclear marker Lamin B1 protein (red) and *ebv-sisRNA-3* (green) in C666-1 cells by combining the Immunohistochemistry and RNAscope RISH analysis. (**E**) RNAscope RISH illustrates the loss of *ebv-sisRNA-3* signals in C666-1 cells upon RNase A treatment. The distance presented by scale bars is indicated. Scale bars represent 10 μm

To validate the existence and cellular localization of *ebv-sisRNA-3*, we performed RNAScope RISH analysis on a total of 24 EBV-positive epithelial cancer samples, including cell lines, patient-derived xenografts and clinical tumor specimens of NPC and EBVaGC. The *ebv-sisRNA-3* was successfully detected in all EBV-positive NPC and EBVaGC samples, while no signal was observed in any of the EBV-negative cells (NP460, NP69, AGS, HK1) (Fig. 2A-C) (Figure S4) (Table S3). Notably, the *ebv-sisRNA-3* transcript was also detected in two latency III LCL cell lines, but not in a Burkitt’s lymphoma cell line, Raji (Figure S5). The co-localization of *ebv-sisRNA-3* with the nuclear marker Lamin B1 in NPC cells C666-1 indicates that *ebv-sisRNA-3* is predominantly localized in the nucleus (Fig. 2D). Moreover, the loss of *ebv-sisRNA-3* signals following RNase A treatment indicates that the signals detected by RNAScope RISH are not derived from the EBV DNA sequence (Fig. 2E). In addition, we also examined the expression of *RPMS1* and *LF3* transcripts in the NPC and EBVaGC samples. While *ebv-sisRNA-3* is derived from the intron of *RPMS1*, *RPMS1* transcripts were also consistently detected in all EBV-positive NPC and EBVaGC cancer cells (Figure S6). Of note, the lytic *LF3* transcript was rarely found in the NPC and EBVaGC primary tumor specimens, but occasionally detected in the cells undergoing lytic replication in the cancer cell lines and xenografts (Figure S7). The findings indicated constitutive nucleus accumulation of *ebv-sisRNA-3* transcripts in all latently infected tumor cells in these two EBV-associated epithelial cancers.

### *ebv-sisRNA-3* forms R-loop at EBV genome in the latently infected tumor cells

**Fig. 3:**
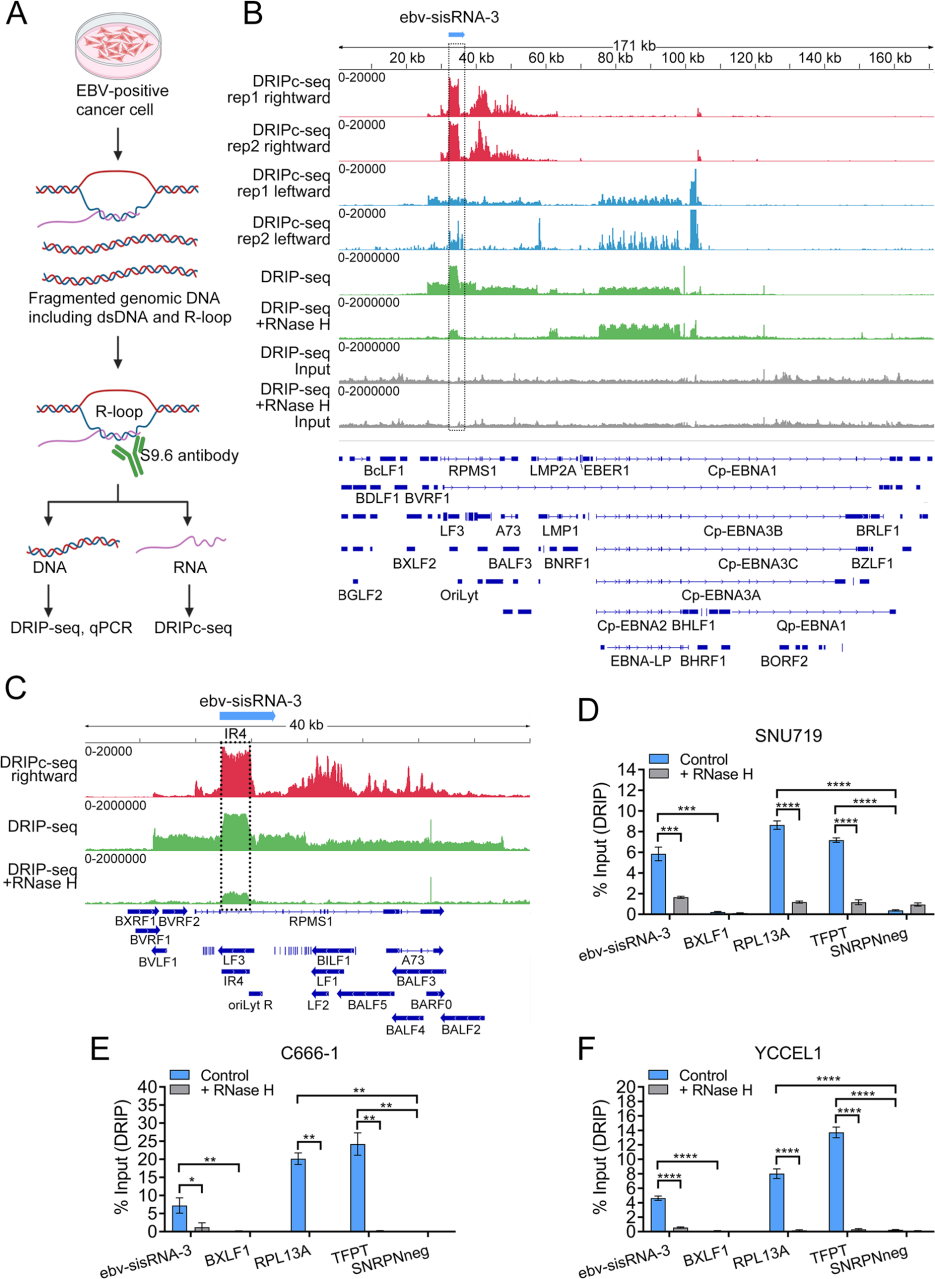
*ebv-sisRNA-3* forms R-loop at EBV genome in the latently infected tumor cells. (**A**) A schematic diagram illustrating the workflow of DRIP-seq and DRIPc-seq. Briefly, genomic DNA containing both dsDNA and R-loop was extracted, fragmented and incubated with S9.6 antibody, while only the R-loop was immunoprecipitated by the S9.6 antibody. Subsequently, DNA and RNA components in R-loop were isolated and applied to DRIP-seq and DRIPc-seq for validation, respectively. (**B**) IGV review of the DRIPc-seq and DRIP-seq of EBV genome from SNU719 cell. The DRIPc-seq is duplicated (rep1 and rep2). DRIPc-seq peaks of rightward and leftward EBV transcripts from the duplicates are shown in track 1–4, respectively. The DRIP-seq peaks from rep1 (with or without RNase H treatment) and their inputs are shown in track 5–8. respectively. (**C**) IGV review of *RPMS1* region from the DRIPc-seq (rightward) and DRIP-seq data in SNU719 cells. (**D-F**) DRIP-qPCR was performed in the *IR4* repeat region of *ebv-sisRNA-3* in C666-1, SNU719, and YCCEL1 cells. The EBV R-loop negative control *BXLF1*, the human positive R-loops foci (*RPL13A*, *TFPT*) and negative foci (*SNRPN*) were tested. DRIP-qPCR was conducted in samples treated with RNase H in parallel. Data is represented as mean ± SD, *N* = 3 technical replicates, *p*-values from two-tailed t-tests are shown: * < 0.05, ** < 0.01, *** < 0.001, **** < 0.0001

As described in previous studies, sisRNAs can modulate gene expression by recruiting functional proteins to target DNA and/or RNA, which are complementary to the sisRNAs themselves [10]. According to the reported sisRNA features, we speculated that the abundant *ebv-sisRNA-3* transcript may also interact with its complementary DNA strand within EBV episome in the latently infected epithelial cancer cells. Its nuclear localization further supports this hypothesis. Additionally, the high GC content in the *IR4* region favor the formation of both R-loop and G-quadruplexes, which could be a reasonable interaction model between *ebv-sisRNA-3* and its parental DNA sequence [47]. Thus, we hypothesize that *ebv-sisRNA-3* may participate in the R-loop and G4 formation within the *IR4* region of the EBV genome. To map the potential R-loops in the EBV genome, we conducted DNA: RNA Immunoprecipitation followed by sequencing (DRIP-seq) and DNA: RNA Immunoprecipitation followed by cDNA conversion and sequencing (DRIPc-seq) using the R-loop antibody S9.6 in the EBV-positive SNU719 cells (Fig. 3A). The DRIP-seq and DRIPc-seq could detect the DNA and RNA components within R-loop structures, respectively. In comparison to the conventional DRIP-seq approach, DRIPc-seq is characterized by strand-specificity and enhanced resolution [43]. As expected, DRIP-seq has revealed the formation of R-loop in *IR4* region within the EBV genome (Fig. 3B and C). By DRIPc-seq, we confirmed the RNA components of this R-loop are identical to the 5’ region of *ebv-sisRNA-3* which binds to upstream of *OriLyt* R (Fig. 3B and C). Interestingly, R-loops formed in adjacent *BART* region that involves with the *RPMS1* transcripts and *BART* miRNAs or their precursors, were also identified by DRIP/DRIPc-seq (Fig. 3B and C).

The presence of R-loops at the *IR4* region and the 5’ region of *ebv-sisRNA-3* was further confirmed through DRIP-qPCR. Notably, R-loops were not detected in the *BXLF1* gene, which served as a R-loop-negative control region within the EBV genome (Fig. 3D and F). Treatment with RNase H, an enzyme that specifically degrades the RNA component of DNA: RNA hybrids, led to a significant reduction in signals at the *IR4* region in both DRIP-seq and DRIP-qPCR assays, providing additional evidence for the authentic formation of R-loop by *ebv-sisRNA-3* (Fig. 3B and F). To validate the reliability of the DRIP/DRIPc-seq, reported R-loop-positive loci *RPL13A* and *TFPT* and -negative loci *SNRPN* in human genome were also examined by the DRIP-qPCR. The R-loop signals were exclusively detected in positive loci, with a marked decrease observed in the presence of RNase H treatment (Fig. 3D and F).

### Endogenous G-quadruplexes in upstream of OriLyt R are associated with* ebv-sisRNA-3*

**Fig. 4:**
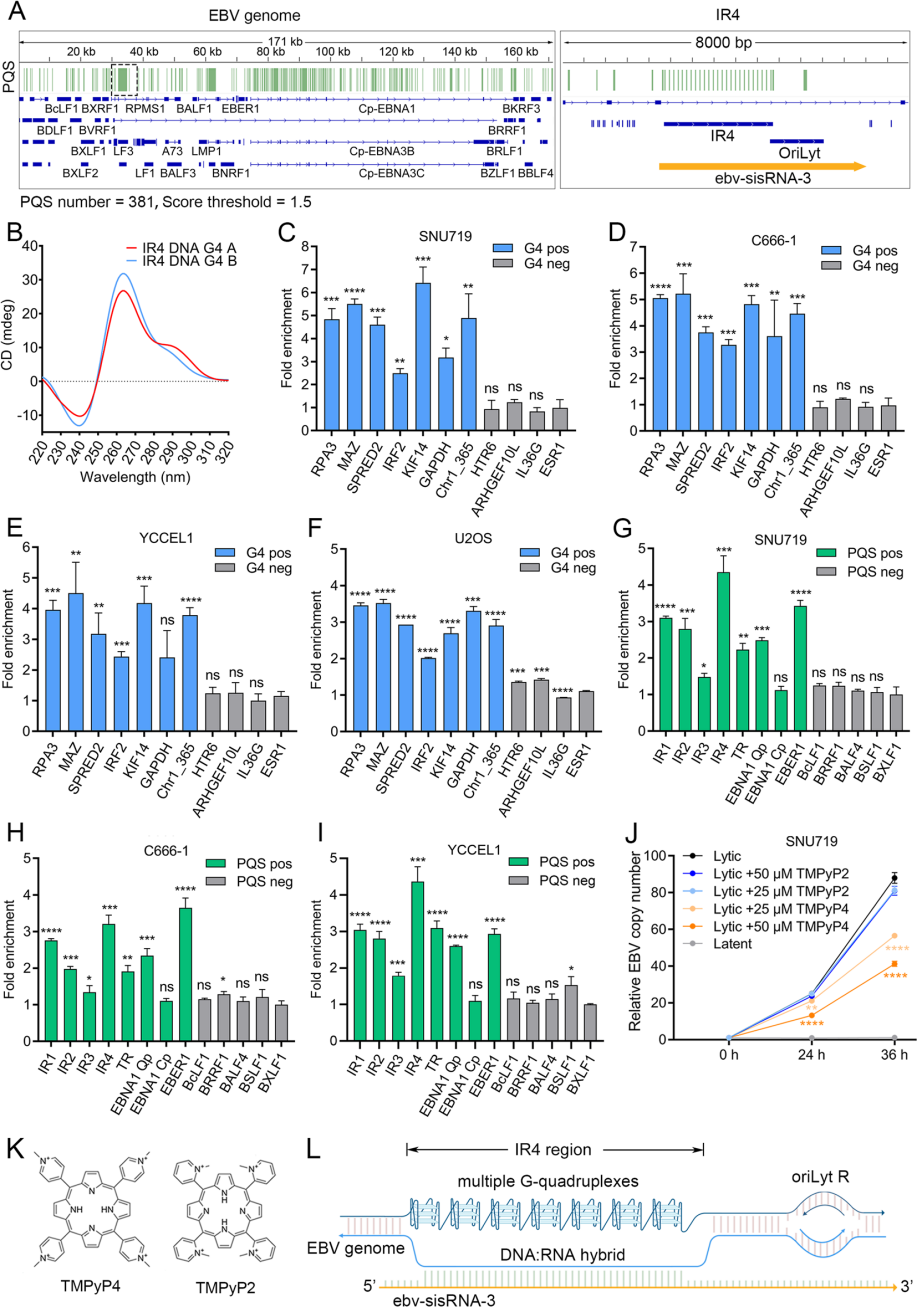
Endogenous G-quadruplexes in *IR4* region are associated with *ebv-sisRNA-3*. (**A**) Locations of PQS in EBV genome. Total of 381 PQS in EBV genome are predicted using G4Hunter (window size = 25, threshold = 1.5). The PQS in *IR4* are shown in right. (**B**) Circular dichroism study for the PQS in *IR4* region. (**C-F**) The G4 fold enrichment in human G4 positive and G4 negative regions were assessed by G4 ChIP-qPCR in EBV-positive (C666-1, SNU719 and YCCEL1) and EBV-negative U2OS cells. (**G-I**) The G4 fold enrichment in PQS-positive and PQS-negative regions of EBV genome was assessed by G4 ChIP-qPCR. (**J**) SNU719 cells were incubated with TMPyP2 or TMPyP4 for 48 h before lytic reactivation by doxycycline. The relative EBV copy number at 0, 24 and 36 h after lytic induction was determined by qPCR assay. Data is represented as mean ± SD in (**C-J**), *N* = 3 technical replicates, *p*-values from two-tailed t-tests (comparing each group with *ESR1* for human genes and *BXLF1* for EBV genes in (C-I), comparing TMPyP4 with TMPyP2 treatment in (**J**)) are shown: ns > 0.05, * < 0.05, ** < 0.01, *** < 0.001, **** < 0.0001. (**K**) Chemical structures of TMPyP4 and TMPyP2 are shown. (**L**) A schematic diagram depicts the G4s and R-loop in *IR4* region associated with *ebv-sisRNA-3*

The high GC content in the *IR4* region, along with the DNA: RNA hybrid formed by the leftward DNA strand and *ebv-sisRNA-3*, suggests the potential for G-quadruplex formation in the single rightward DNA strand of this region. By G4Hunter with a stringent threshold, we predicted potential G-quadruplex sequences (PQS) in *IR4* region and also a total of 381 PQS in the EBV genome [48] (Fig. 4A). To investigate the formation of G4 structures in the *IR4*, we analyzed the circular dichroism (CD) signature of two PQS from the *IR4* region. Both PQS exhibited a positive absorption peak at approximately 260 nm, a negative absorption peak at around 240 nm and a weak positive absorption peak at 280–290 nm. These signatures indicate that the *IR4* PQS form parallel and hybrid G-quadruplexes in vitro [49] (Fig. 4B). Subsequently, we synthesized and purified a G4-specific antibody BG4, which demonstrated high specificity for G4 sequences in vitro [50] (Figure S8). To confirm that the BG4 antibody is capable of detecting endogenous G4 structures, we initially evaluated previously reported human G4 sites using G4 ChIP-qPCR. Almost all reported G4 sites were successfully detected in the three EBV-positive cell lines and an EBV-negative U2OS cell line (Fig. 4C-F). We further assessed the endogenous G4 formation in *IR4* region by G4 ChIP-qPCR. As expected, G4 ChIP-qPCR detected 3–5-fold enrichment of G4 structures in the *IR4* region across all three EBV-positive cell lines compared to PQS-negative control regions in the EBV genome. (Figs. 4G-I).

We also assessed a set of PQS within the EBV genome, including PQS in other internal repeats (*IR1*,* IR2*,* IR3*), terminal repeat (*TR*) and PQS around the promoter of *EBNA1* and *EBER1*. These selected PQS were either located in high GC content region, which favored the G4 formation, or in the promoters of important latent EBV genes. The CD spectroscopy analysis indicated that these PQS could form parallel and/or hybrid G-quadruplexes in vitro (Figure S9). Additionally, more than 2-fold of G4 enrichment was detected in *IR1*, *IR2*, *TR*, *EBNA1* Q promoter (Qp) and *EBER1* promoter by G4 ChIP-qPCR, suggesting that endogenous G4s are also formed in these regions (Figs. 4G-I). The widespread existence of endogenous G4s in EBV genome implies G4 may play regulatory roles during EBV latent stage.

Since the *IR4* G4 is localized to the upstream of *OriLyt*, its function might be associated with EBV lytic replication. To investigate whether the G4s formed in *IR4* could affect the lytic DNA replication, we incubated the SNU719 cells with a G4 stabilizer, TMPyP4, for 48 h before lytic induction. The lytic DNA replication was significantly suppressed by the TMPyP4 with a dose dependent manner. While its structural isomer TMPyP2, which has little G4 affinity [51, 52], showed no inhibitory effect on EBV lytic DNA replication (Fig. 4J **and K**). It indicated the TMPyP4 mediated inhibitory effect on lytic DNA replication was G4-specific. TMPyP4 may suppress EBV lytic DNA replication through targeting the G4 in *IR4*, which is adjacent to upstream of *OriLyt* R and possibly play a functional role in initiating viral genome replication from *OriLyt* R. To conclude, our results indicate that endogenous G4 and R-loop associated with *ebv-sisRNA-3* are formed in *IR4* region and may act as a suppressor for EBV lytic replication (Fig. 4L).

## Discussion

In this study, we identified and characterized a new EBV-encoded sisRNA, *ebv-sisRNA-3* in the EBV-infected epithelial cells. Similar to the *ebv-sisRNA-1* and *ebv-sisRNA-2* reported previously, *ebv-sisRNA*-3 is derived from EBV intronic sequences during the splicing process of the latent viral transcripts and is stably accumulated in the infected cells. The biogenesis of *ebv-sisRNA-1* and *ebv-sisRNA-2* was shown to be generated from the W-repeats in the *IR1* region of the EBV genome in the EBV latency III infected B cells [15]. In contrast, *ebv-sisRNA-3* is derived from the *BART* intron 1 region harboring *IR4* repeats and is detected in both epithelial cells with latency I/II infection and B-cells with latency III infection, but not in Raji, a Burkitt’s lymphoma cell line. Previous study has revealed that *ebv-sisRNA-1* and *ebv-sisRNA-2* interact with human HNRNPL and HNRNPD proteins, probably implicating in the pro-cancer-related phenotypes [14]. Here we found that *ebv-sisRNA-3* contributes to the formation of R-loop and G4 and structures in the EBV episome. These secondary nucleic acid structures may play a functional role of *ebv-sisRNA-3* in EBV latency and warrant further investigation.

Traditionally, EBV-encoded *BARTs* are classified into two groups of non-coding RNAs: the *BART* miRNAs derived from introns and the alternatively spliced polyadenylated lncRNAs. A total of 44 viral miRNAs derived from *BARTs* have been identified, and these miRNAs play various roles in the transformation, proliferation, and immune evasion of EBV infected cells [53]. The polyadenylated *BART* lncRNAs are implicated in modulating the transcription of the interferons and cell adhesion genes [54]. In this study, we introduced a third group of *BART* non-coding RNA: *ebv-sisRNA-3*, which is generated from the introns of *BART*. Unlike the other *BART* lncRNAs, *ebv-sisRNA-3* is not polyadenylated and has not been detected in other EBV transcriptome studies using standard poly(A)-captured RNA sequencing. Our finding expands the understanding of the complexity of non-coding transcripts in *BART* region. The *ebv-sisRNA-3* contains the repeated sequences exceeding 2000 kb in length and extremely high GC content. Thus, ectopic transfection and knockdown of *ebv-sisRNA-3* are technical challenges, limiting the functional studies of *ebv-sisRNA-3* in the EBV-infected cells.

Rennekamp and Lieberman have reported that the R-loop formed by *BHLF1* at the OriLyt L during early lytic replication is essential for lytic replication initiation [26]. Here we revealed that a R-loop is also formed upstream of *OriLyt* R of the EBV episome by *ebv-sisRNA-3* during latent stage. Although the lytic transcript *LF3* is complementary to *ebv-sisRNA-3* and contains the *IR4* region, we excluded the possibility of the R-loop being formed by *LF3* for two reasons: first, RNAscope RISH assay and RNA-seq have demonstrated that *LF3* is rarely expressed in latent cells; second, the strand-specific DRIPc-seq confirm the *LF3* was not involved in the R-loops in SNU719 cells. Notably, R-loops are identified across nearly the entire *BART* region in our study for the first time. These newly identified R-loops may suggest a novel R-loop-mediated mechanism regulating *BART* transcript expression across the *BART* region.

The G4s formed in the EBV genome region with *ebv-sisRNA-3*-associated R-loop were demonstrated in our G4 ChIP assays. These G4 and R-loop structures may explain the stability of *ebv-sisRNA-3* in the nucleus of infected cells. The presence of G4s in the *IR4* region was identified in both NPC and GC cells, indicating that their formation is ubiquitous among EBV-positive epithelial cells. G4s are functional elements in the genome, and their formation is positively associated with increased transcriptional activity [21]. G4 motifs are also enriched in replication origins, suggesting that G4s regulate initiation of DNA replication [29]. Given that the G4 in *IR4* region is located near the lytic replication origin of EBV, we speculate that it may play a regulatory role in lytic replication. Consistent with this speculation, treatment with G4 ligands suppresses the copy number of lytic viral DNA by ~ 50%. We thus propose that the *ebv-sisRNA-3*-associated R-loop and G4 structures at upstream of *OriLyt* R may serve as a suppressor for the initiation of lytic replication. Further investigation is needed to elucidate the detailed biological roles of the *ebv-sisRNA-3*-associated R-loop and G4 structures in EBV episome and its interactions with cellular components.

## Conclusions

In this study, we discovered and comprehensively characterized a novel EBV-encoded ncRNA, *ebv-sisRNA-3*. Our findings demonstrated that *ebv-sisRNA-3* binds to EBV genome *in trans*, forming a unique G4/R-loop structure upstream of *OriLyt* R, which may suppress the initiation of lytic replication. Additionally, we revealed the locations of R-loop within the EBV episome and identified multiple endogenous G-quadruplexes within EBV genome during the latent stage.

## Electronic supplementary material

Below is the link to the electronic supplementary material.

Supplementary Material 1

## Data Availability

Not applicable.

## Abbreviations

EBV: Epstein-barr virus
NPC: Nasopharyngeal carcinoma
EBVaGC: EBV-associated gastric cancer
sisRNA: Stable intronic sequence RNA
lncRNA: Long non-coding RNA
miRNA: microRNA
OriLyt: Lytic DNA replication origin
IR4: Internal repeat
DRIP-seq: DNA: RNA immunoprecipitation followed by sequencing
DRIPc-seq: DNA: RNA immunoprecipitation followed by cDNA conversion and sequencing
PQS: Potential G-quadruplex sequences
CD: Circular dichroism
