# Supplementary material for: ebv-sisRNA-3 contributes to the formation of G4-associated R-loop upstream of EBV lytic replication origin in latently infected cells
Source: Cell Biosci. 2025 Jun 27;15:91. doi: 10.1186/s13578-025-01437-3 (PMC12203722; doi:10.1186/s13578-025-01437-3)
Supplement: Supplementary file 1 — Supplementary Material 1 [file 13578_2025_1437_MOESM1_ESM.pdf]

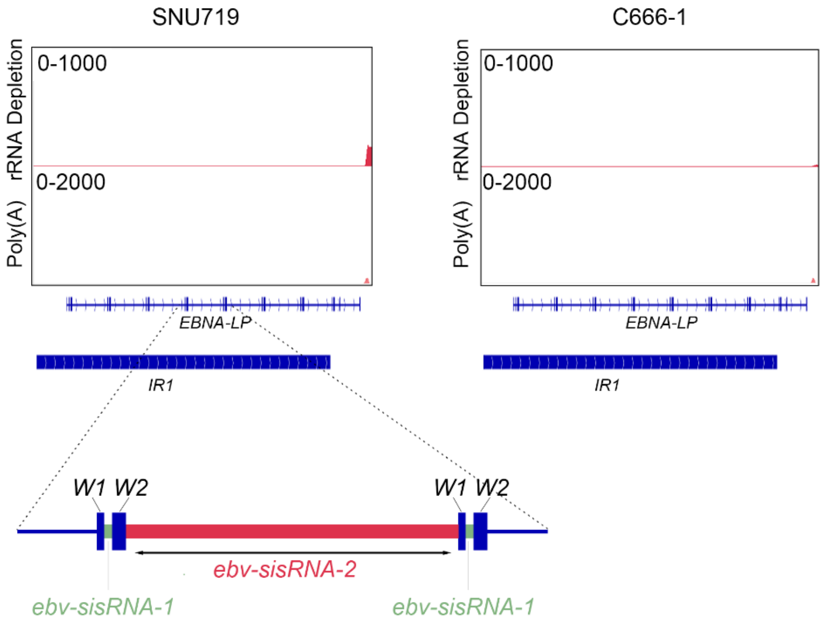

Figure S1. IGV view for *ebv-sisRNA-1* and *ebv-sisRNA-2* in EBV-positive NPC and GC.

IGV view for rightward peak of *ebv-sisRNA-1* and *ebv-sisRNA-2* from RNA sequencing on both ribosomal RNA-depleted and poly(A)-captured RNA libraries of SNU719 (left panel) and C666-1 (right panel). *ebv-sisRNA-1* and *ebv-sisRNA-2* are located in the introns between W1 and W2 exons of *EBNA-LP*. Both RNA-seq results indicated that *ebv-sisRNA-1* and *ebv-sisRNA-2* are not expressed in SNU719 and C666-1 cells.

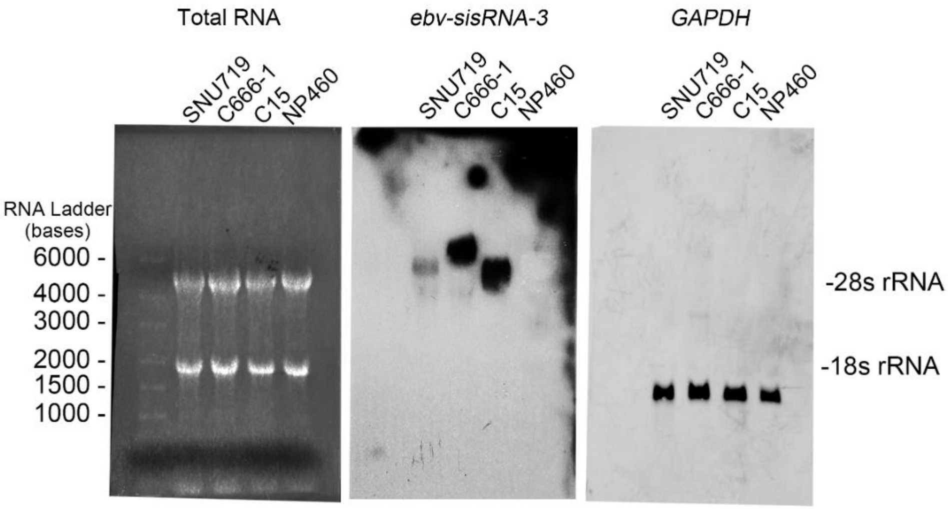

Figure S2. Uncropped figures of Northern blot experiments in Figure 1C.

The left panel showed the blot with total RNA and RNA ladder (pre-stained by GelRed

during agarose gel electrophoresis) imaged after capillary transfer and UV-crosslinking. The middle and right panels showed the bands of *ebv-sisRNA-3* and *GAPDH* from the same blot with left panel, respectively.

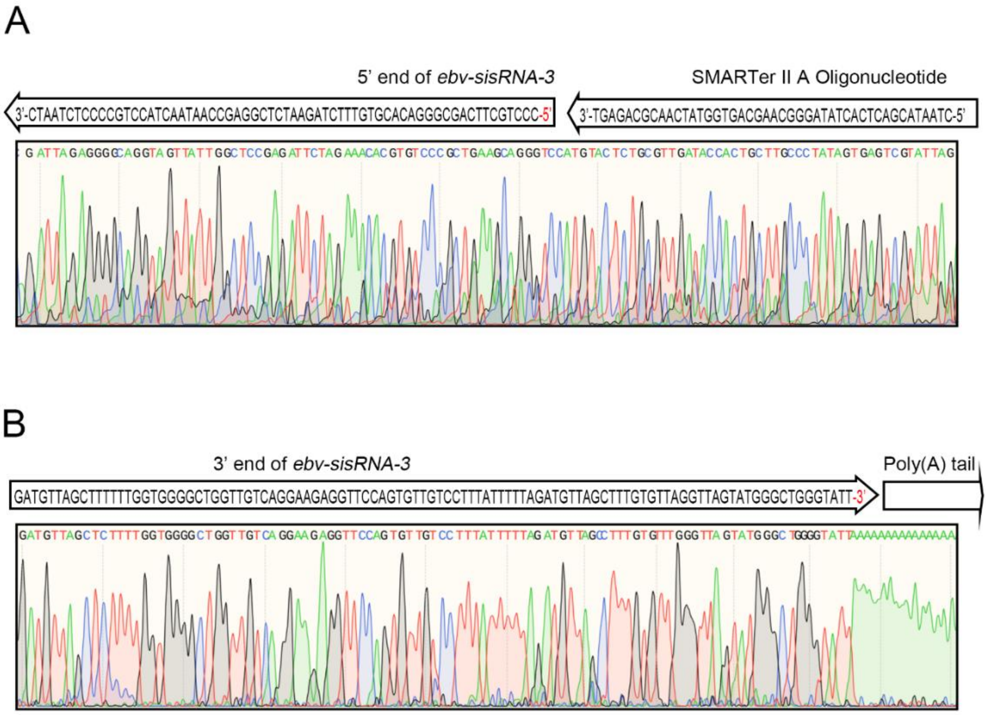

Figure S3. Sanger sequencing for 5' and 3' RACE.

(A) The sequence with SMARTer II A Oligonucleotide adding to 5' end of *ebv-sisRNA-3* sequence was identified by sanger sequencing. The sequencing result was presented as reverse complementary. Several random bases between SMARTer II A Oligonucleotide and 5' end of *ebv-sisRNA-3* were generated during the RACE reaction. (B) The sequence with Poly(A) tail adding to 3' end of *ebv-sisRNA-3* sequence was identified by Sanger sequencing.

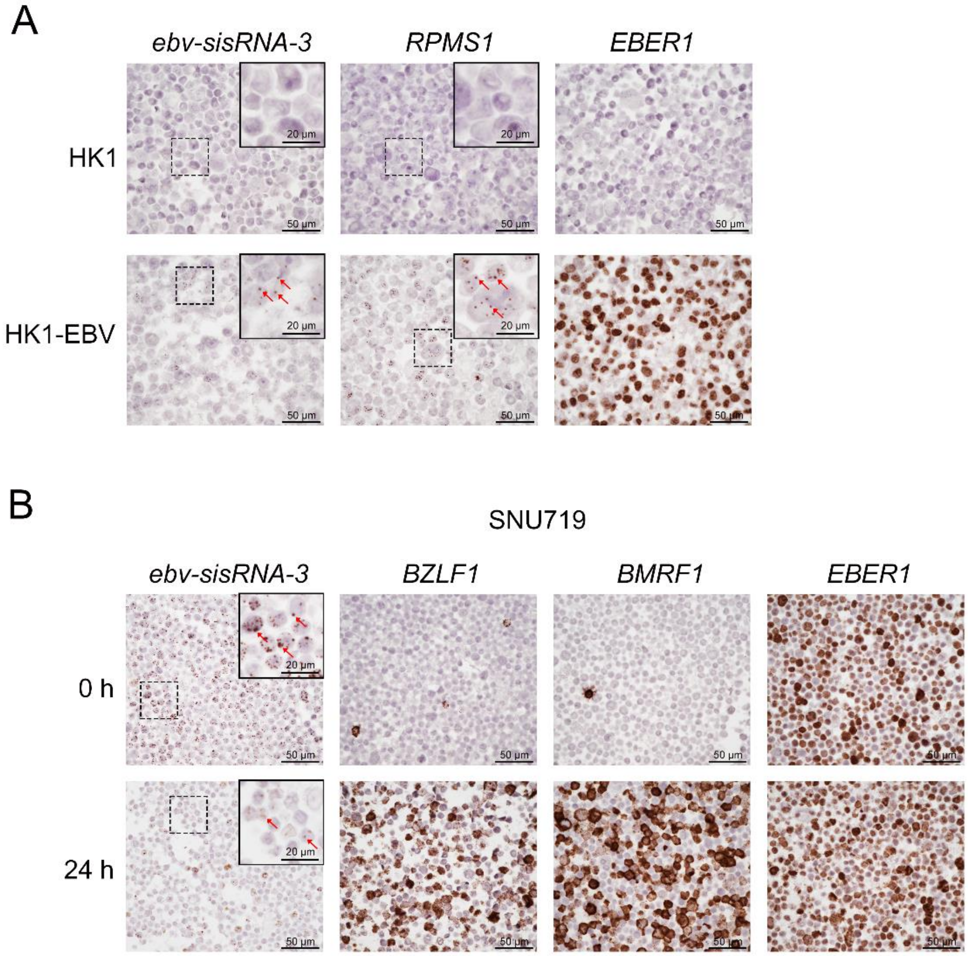

Figure S4. *ebv-sisRNA-3* expression in EBV-infected epithelial cells. (A) RNAscope RISH analysis of *ebv-sisRNA-3*, *RPMS1* and *EBER1* transcripts in an EBV-negative NPC cell line (HK1) and an artificially constructed EBV-positive NPC cell line (HK1-EBV). Representative signals for *ebv-sisRNA-3* and *RPMS1* are indicated with red arrows. (B) RNAscope RISH analysis of *ebv-sisRNA-3* in EBV-positive SNU719 cells after lytic reactivation. RNAscope RISH analysis of *ebv-sisRNA-3*, *BZLF1*, *BMRF1* and *EBER1* in the Dox-treated stably transfected SNU719 with sgRNA3, HA-dCas9-2A-EGFP, and inducible 3xFLAG-PUFa-p65HSF1 transactivator at 0 and 24 hours. In our previous study, expression of EBV lytic genes, BZLF1, BRLF1 and BGLF4 were detected in the Dox-treated control stably transfected SNU719 with sgCtl, HA-dCas9-2A-EGFP, and 3xFLAG-PUFa-p65HSF1 (ref. 40). The signals of *ebv-sisRNA-3* are indicated with red arrows.

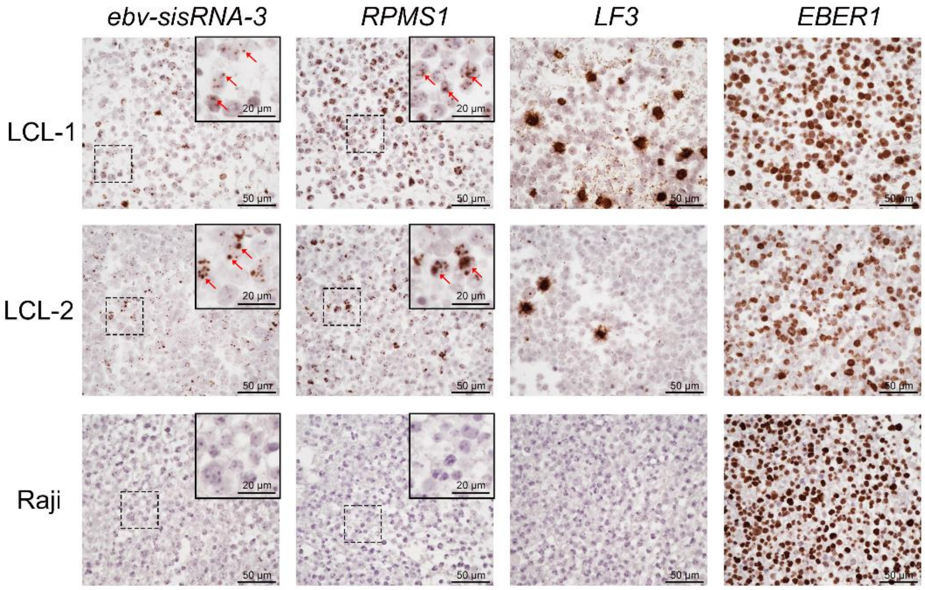

Figure S5. RNAscope RISH analysis for *ebv-sisRNA-3, RPMS1*, *LF3* and *EBER1* in two LCL (LCL-1, LCL-2) and an EBV-positive Burkitt lymphoma cell line (Raji). Representative signals of *ebv-sisRNA-3* and *RPMS1* are indicated with red arrows.

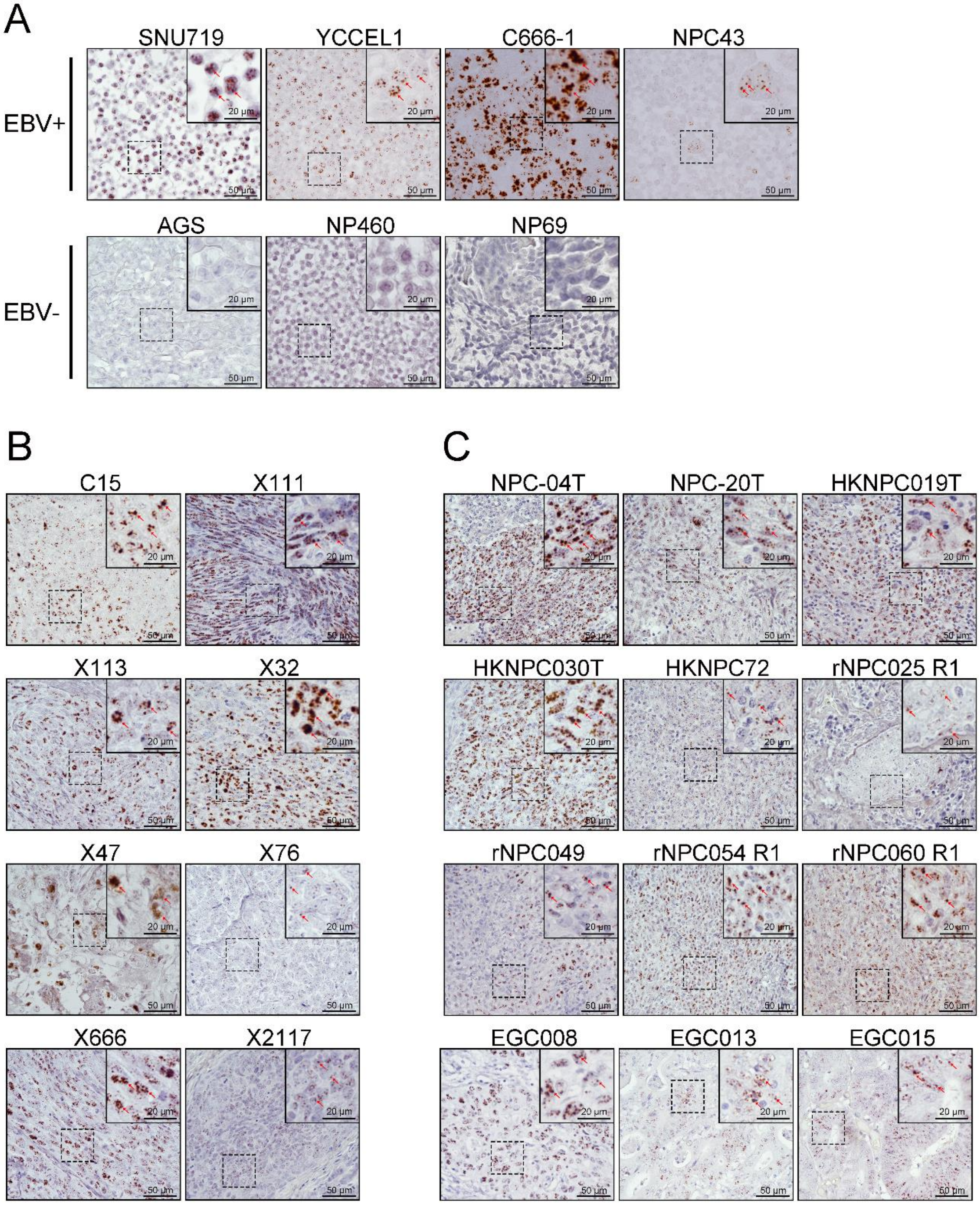

Figure S6. RNAscope RISH analysis for *RPMS1*.

RNAscope RISH analysis for *RPMS1* in both EBV-positive and EBV-negative cell lines (A), NPC xenografts (B) and NPC and EBVaGC patient specimens (C) are shown. The enlarged image of the area within the dashed box is displayed in the upper right corner of each picture. Signals of *RMPS1* are indicated with red arrows.

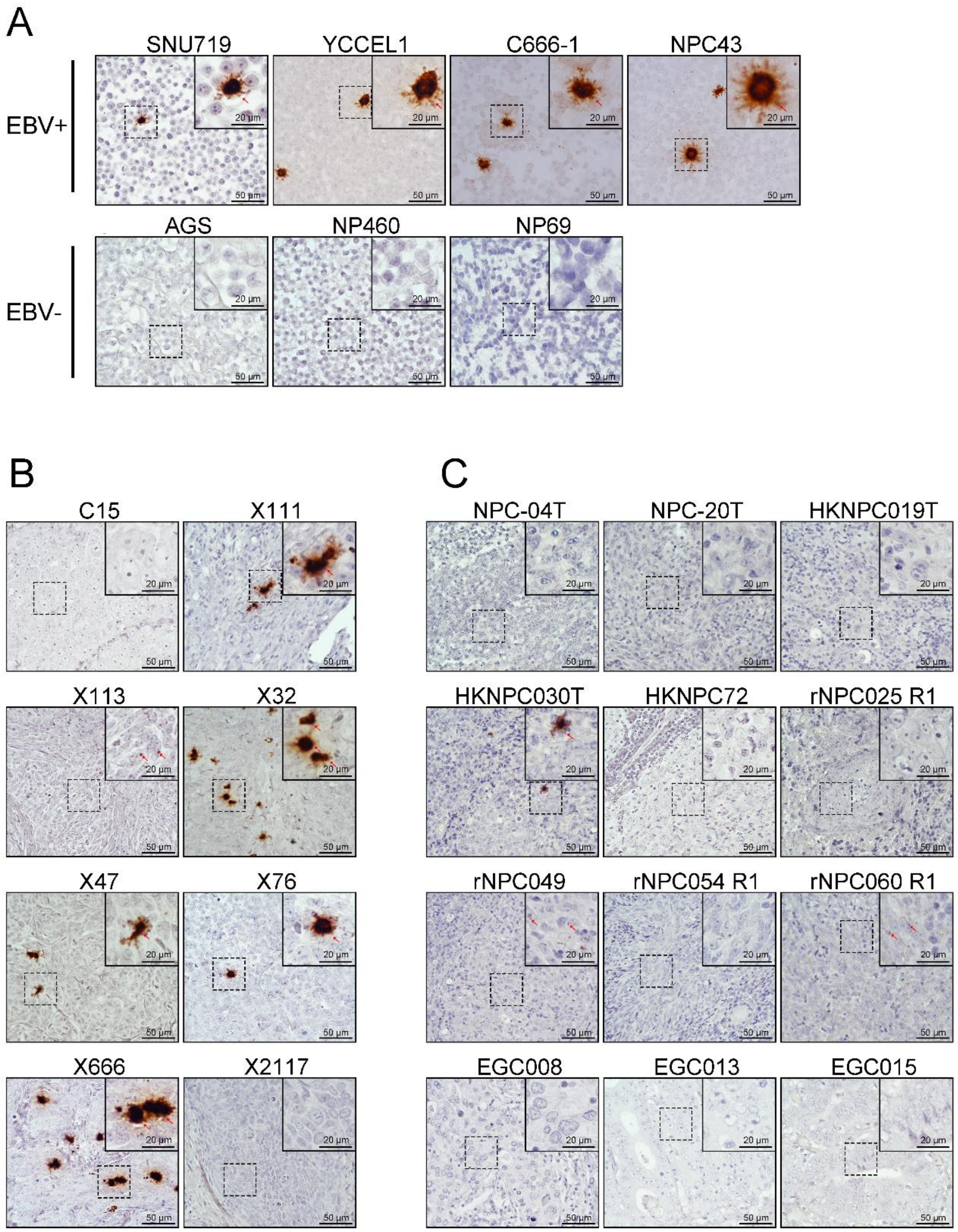

Figure S7. RNAscope RISH analysis for *LF3*.

RNAscope RISH analysis for *LF3* in both EBV-positive and EBV-negative cell lines (A), NPC xenografts (B) and NPC and EBVaGC patient specimens (C). The enlarged image of the area within the dashed box is displayed in the upper right corner of each picture. Signals of *LF3* are indicated with red arrows.

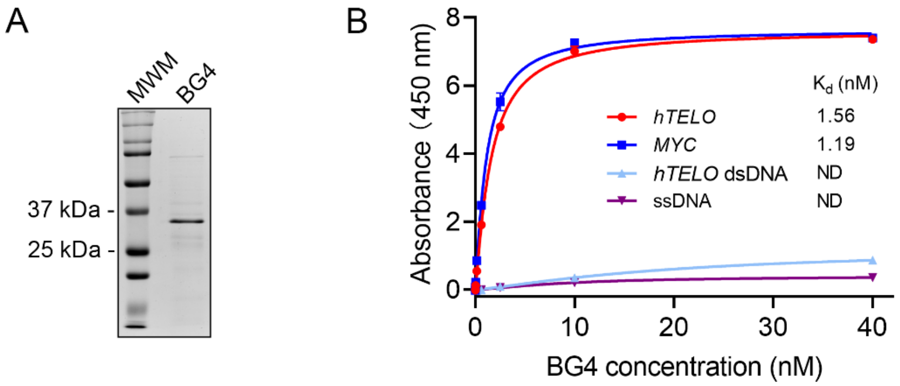

Figure S8. Assessment of the G4 antibody BG4.

(A) The 30 kDa BG4 antibody (lane 2) was purified and stained with Coomassie in SDS-PAGE gel. (B) The affinity between BG4 and G4 oligos (*hTELO*, *MYC*), double strand DNA (*hTELO* dsDNA), or non-G4 oligo (ssDNA) were assessed by ELISA assay. The *hTELO* and *MYC* G4 sequences exhibited high affinity with BG4 antibody, with Kd=1.56 nM and Kd =1.19 nM, respectively. While the double strand *hTELO* dsDNA and ssDNA showed no detectable binding (ND) with BG4.

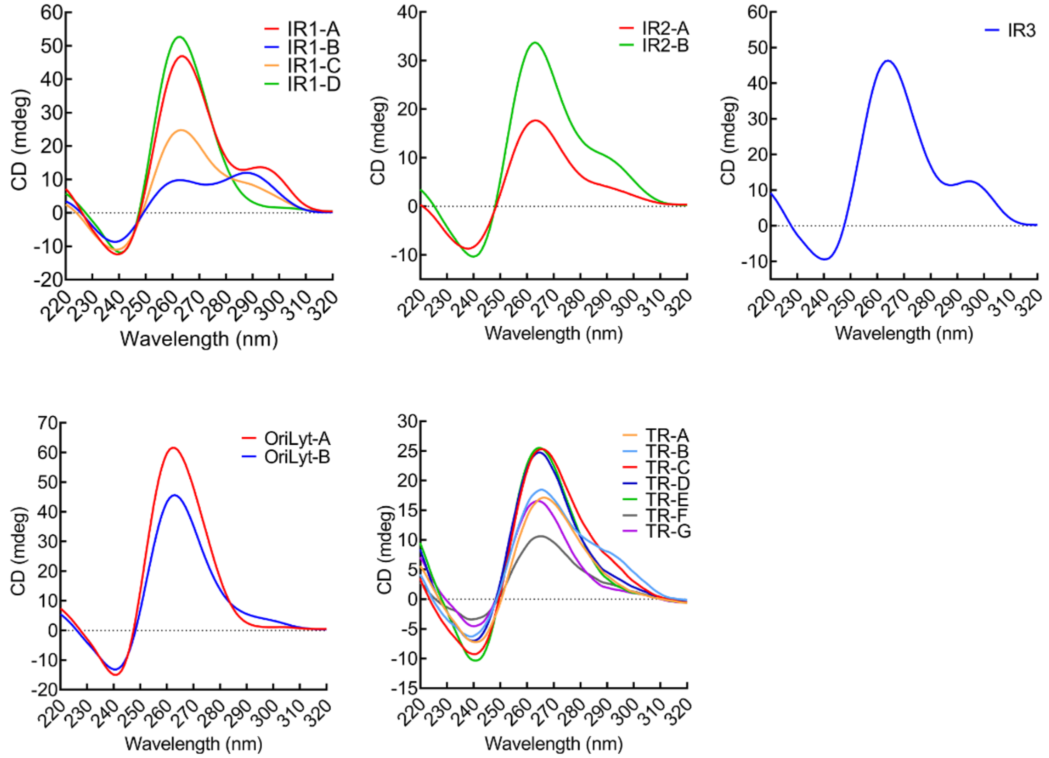

Figure S9. CD spectra for PQS in EBV genome.

The 5 µM of PQS oligos of internal repeats (*IR1*, *IR2*, *IR3*), terminal repeat (*TR*), PQS in *OriLyt*, and PQS around the promoter of *EBNA1* and *EBER1* were tested under 100 mM

KCl condition. Sequences for these oligos are listed in **Table S5**. The CD ellipticity (measured in millidegrees (mdeg)) from 220 to 320 nm at 1 nm interval were accumulated.

Table S1. Template and primers for *in vitro* transcription.

Name	Sequence (5'-3')
T7-*IR4* template DNA	TAGGGAGAGCGGGGCAGCGGACCCAGCGGACCCGGTGGGCC ACCCGGCCGCCCCCCGAGCTCCAGGGCCGGAACCCCGGACCC GGCTGCA
T7-*IR4* forward	TAATACGACTCACTATAGGGAGAGCGGGGCAGC
T7-*IR4* reverse	TGCAGCCGGGTCCGGG
T7-*GAPDH* forward	TAATACGACTCACTATAGGGATGATGTTCTGGAGAGCC
T7-*GAPDH* reverse	TCACCACCATGGAGAAGGCTGG

Table S2. Primers for RACE-PCR.

Name	Sequence (5'-3')
ebv-sisRNA-3 5' RACE 1st round	GATTACGCCAAGCTTCCGGAACCCCGGACCCGG CTGCA
ebv-sisRNA-3 5' RACE 2nd round	GATTACGCCAAGCTTGGGTCCTGAGACCCAAAG CGACAGGGGG
ebv-sisRNA-3 3' RACE 1st round	CAGAGGAGGGGGTGTTGGTGTGCAA
ebv-sisRNA-3 3' RACE 2nd round	TGACATTAGACAGTGGACACCGGGCT

Table S3. Staining score of RNAScope for *ebv-sisRNA-3* and *RPMS1*.

		Staining score	
		ebv-sisRNA-3	*RPMS1*
NPC cell	C666-1	+++	++++
	NPC43	+	+
Immortalized NP cells	NP69	0	0
	NP460	0	0
NPC Xenograft	C15	++	+++
	C17	+	+
	X113	++	++++
	X2117	+++	+++
	X23	++	++++
	X32	++	++++
	X47	++	++++
	X666	+++	++++
NPC Specimen	HKNPC72	++	+++
	HKNPC019T	++	+++
	HKNPC030T	++	++++
	NPC-04T	++	++++
	NPC-20T	++	+++
	rNPC025 R1	+++	++
	rNPC049	++	+++
	rNPC054 R1	++	+++
	rNPC060 R1	++	+++
GC cell	SNU719	++	+++
	YCCEL1	++	+++
	AGS	0	0
GC Specimen	EGC013	++	+++
	EGC008	++	++++

EGC015	++	+++

The staining for *ebv-sisRNA-3* and *RPMS1* were categorized into five grades: 0, +, ++, +++, and ++++ according to the following criteria: 0, no staining or less than 1 dot to every 10 cells; +, 1–3 dots/cell; ++, 4–10 dots/cell and very few dot clusters; +++, >10 dots/cell and less than 10% positive cells have dot clusters; ++++, >10 dots/cell and more than 10% positive cells have dot clusters.

Table S4. Primers for DRIP-qPCR.

Name	Sequence (5'-3')
IR4 forward	CACCGGGTCCGCTGGGT
IR4 reverse	AGGGCCGGAACCCCGGA
BXLF1 forward	CGGTAAACGTGGCCAAAACTT
BXLF1 reverse	ATGATCGCCATTTGCTGTCG
RPL13A forward	AGGTGCCTTGCTCACAGAGT
RPL13A reverse	GGTTGCATTGCCCTCATTAC
TFPT forward	TCTGGGAGTCCAAGCAGACT
TFPT reverse	AAGGAGCCACTGAAGGGTTT
*SNRPN*neg forward	GCCAAATGAGTGAGGATGGT
*SNRPN*neg reverse	TCCTCTCTGCCTGACTCCAT

Table S5. Sequences for CD spectra.

Name	Sequence (5'-3')
IR4 DNA G4 A	GGGGGGTGGCCGGCTGCAGCCGGGTCCGGG
IR4 DNA G4 B	GGGGGGCGGCCGGGTGGCCCACCGGG
IR1-A	GGGGGACGGGGAGGGGGGGAGGCTGGGG
IR1-B	GGGACCGGGTGCTGGGACCTCGGG
IR1-C	GGGCCGGTGGGGGGATCCGGGCCACTCGGG
IR1-D	GGGGCGGGAGGGGGCTGGG
IR2-A	GGTCCTGGGGCAGCCGGGGTTCCTGG
IR2-B	GGGTGGGGGGTGGCCCCGCTGGG
IR3	GGGGCAGGAGGGGCAGGAGGGGCAGGAGGG
IR4-A	GGGGGGTGGCCGGCTGCAGCCGGGTCCGGG
IR4-B	GGGGGGCGGCCGGGTGGCCCACCGGG
TR-A	GCCGCTCTGTGCGGGGGGGCTGGGGGGCCGCGGGGGAAGGCCACG
TR-B	CGGGCGGGCGGCGGGGGGTCGGGGTCCGCGGGCTCCGGGGGCTGC GGGCGGTGGATGGCG
TR-C	CGGGGATCGGGGGGGTCGGGGGGCGCCGCGCGGGC
TR-D	GTGATGAGGGGGCAGGGTCGCAGGGGGTGTGTCTGGTGGGGGCGGG AGCGGGGGGCGGCGCGGGAG
TR-E	GGAGGGTAGAATGACAGGGGGCGGGGACAGAGAGGCGGTCG
TR-F	GGGGGCTTGGCTGGCGCGGCCGGGGG
TR-G	GGGGGGCGGGGAGCGGGCAATGGAGCG
OriLyt A	GGGGGGGGTAGGGGGGGG
OriLyt B	GGGTGCTGGGGTGGGGGATGGG

Table S6. Oligos for BG4 ELISA.

Name	Sequence
h*TELO*	5'-Biotin-GG(TTAGGG)$_4$TTAG-3'
MYC	5'-Biotin-ATGGGGAGGGTGGGGAGGGTGGGGAAGGTG-3'
h*TELO* dsDNA	5'-Biotin-GG(TTAGGG)$_4$TTAG-3'/3'-C(AATCCC)$_4$AAT-5'
ssDNA	5'-Biotin-GACATAGTGCGTGAGCG-3'

Table S7. Primers for G4 ChIP-qPCR.

Name	Forward sequence (5'-3')	Reverse sequence (5'-3')

RPA3	CGGAAGTTGACAGATACAGGG	GATCGCAGAAAGGTAGTCTCAG
MAZ	ACTCAGCGCAGGATTGTAAATA	CCTCATGCTTCGGCTTCC
SPRED2	AACAGGAGGAGGAAGTAGGG	TTTCGGTCGCAAGTAGGAAG
IRF2	TGAAAGCCCGTCAGTTGAATAA	GCTTTCGATCTGGACTGTTCTC
KIF14	CGGTAGCCGTCTCTGAATG	CTTTAGCAGAACCCGAGGAG
GAPDH	GCTACTAGCGGTTTTACGGGCG	TGCGGCTGACTGTCGAACAGG
Chr1_365	TCTGGCGGCCGCTATTG	GCGGACACGGTTTGGATACTA
HTR6	GGCGATTTGTCCAATATTTCCC	CTGTGACCTGCCCTTATCC
ARHGEF10L	TGCCAAGTTACTCTCAGTTCTG	AGCCAAACCTCCAAGAACAA
IL36G	GCCCACCTCTTTACTTCCTTA	AACACTCTTTCAGCTCCATCC
ESR1	GAAACAGCCCCAAATCTCAA	TTGTAGCCAGCAAGCAAATG
TMCC1	GTGGTACACTGCCTACAGTATT	GTATAACGCCTGGGCTATGT
IR1	CGAGCCTGCTGTCTCAGG	GACTCTGCCAACAGAGACCC
IR2	GGGACACTGCACTACCGCCA	CCGGAGCGCCAGGAACCC
IR3	GGCTCAGGATCAGGGCCAAGACA	CTCCTCCTGCTCCTGCCCC
IR4	CACCGGGTCCGCTGGGT	AGGGCCGGAACCCCGGA
TR	GTGGATGGCGGCGGACGTT	CTCATCACGGTCACGCATGGCTG
EBNA1(Qp)	GTGACCACTGAGGGAGTGTT	TATTACCCGCCATCCGGTAG
EBNA1(Cp)	GGACCTTAGAGGTGGAGCAAC	TTGCGTAAGCAAGGCGTAAT
EBER1	GTAAAACACCGACCGCGCCA	ACAGACTACGTCACCGTGACGG
BcLF1	AACTAAGCCCGGTGTCGATG	TGGGACCCCAAATGTTAGCC
BRRF1	AATTGTGGGTGGATTCGTGC	GGGATTTCATAAAGGCCGGG
BALF4	GTTCTCGATGAGGGAGGTGTT	AGTCCGGCAACGAGATCCA
BGLF2	GGGTTCCATTGATGTAGACGG	TCAGATAGCCGGACCTCCTAA
BKRF3	GCTTCTCCCCGACTTATGGC	TCGGACACAGGCAATAACGG
BSLF1	CCCCAAAAATAGTAAGCAGCCG	CGCATCTACCGCGTTAACATC
BXLF1	CGGTAAACGTGGCCAAAACTT	ATGATCGCCATTTGCTGTCG

Table S8. Primers and probes for EBV DNA copy number analysis.

Name	Sequence (5'-3')
EBV BamHI-W (Bam-W) fragment forward	CCCAACACTCCACCACACC
EBV BamHI-W (Bam-W) fragment reverse	TCTTAGGAGCTGTCCGAGGG
EBV BamHI-W (Bam-W) fragment probe	5'(FAM)CACACACTACACACACCCACCCGTCT C(TAMRA)3'
LEPTIN forward	CAGTCTCCTCCAAACAGAAAGTCA
LEPTIN reverse	GTCCATCTTGGATAAGGTCAGGA
